# Prevalence of uncoupling protein one genetic polymorphisms and their relationship with cardiovascular and metabolic health

**DOI:** 10.1371/journal.pone.0266386

**Published:** 2022-04-28

**Authors:** Petros C. Dinas, Eleni Nintou, Maria Vliora, Anna E. Pravednikova, Paraskevi Sakellariou, Agata Witkowicz, Zaur M. Kachaev, Victor V. Kerchev, Svetlana N. Larina, James Cotton, Anna Kowalska, Paraskevi Gkiata, Alexandra Bargiota, Zaruhi A. Khachatryan, Anahit A. Hovhannisyan, Mariya A. Antonosyan, Sona Margaryan, Anna Partyka, Pawel Bogdanski, Monika Szulinska, Matylda Kregielska-Narozna, Rafał Czepczyński, Marek Ruchała, Anna Tomkiewicz, Levon Yepiskoposyan, Lidia Karabon, Yulii Shidlovskii, George S. Metsios, Andreas D. Flouris

**Affiliations:** 1 FAME Laboratory, Department of Physical Education and Sport Science, University of Thessaly, Trikala, Greece; 2 Faculty of Education Health and Wellbeing, University of Wolverhampton, Walsall, West Midlands, United Kingdom; 3 Laboratory of Gene Expression Regulation in Development, Institute of Gene Biology, Russian Academy of Sciences, Moscow, Russia; 4 Department of Biology and General Genetics, Sechenov First Moscow State Medical University (Sechenov University), Moscow, Russia; 5 L. Hirszfeld Institute of Immunology and Experimental Therapy, Polish Academy of Sciences, Wrocław, Poland; 6 Royal Wolverhampton NHS Trust, New Cross Hospital, Wolverhampton, United Kingdom; 7 Institute of Human Genetics, Polish Academy of Sciences, Poznań, Poland; 8 Department of Endocrinology and Metabolic Diseases, Medical School, Larissa University Hospital, University of Thessaly, Larissa, Greece; 9 Institute of Molecular Biology, National Academy of Sciences of the Republic of Armenia, Yerevan, Armenia; 10 Department of Treatment of Obesity, Metabolic Disorders and Clinical Dietetics, Poznan University of Medical Sciences, Poznań, Poland; 11 Department of Endocrinology, Metabolism and Internal Medicine, Poznan University of Medical Sciences, Poznań, Poland; 12 Department of Bioengineering, Bioinformatics and Molecular Biology, Russian-Armenian University, Yerevan, Armenia; 13 Department of Nutrition and Dietetics, School of Physical Education, Sport Science and Dietetics, University of Thessaly, Trikala, Greece; King Saud University, SAUDI ARABIA

## Abstract

Contribution of *UCP1* single nucleotide polymorphisms (SNPs) to susceptibility for cardiometabolic pathologies (CMP) and their involvement in specific risk factors for these conditions varies across populations. We tested whether *UCP1* SNPs A-3826G, A-1766G, Ala64Thr and A-112C are associated with common CMP and their risk factors across Armenia, Greece, Poland, Russia and United Kingdom. This case-control study included genotyping of these SNPs, from 2,283 Caucasians. Results were extended via systematic review and meta-analysis. In Armenia, GA genotype and A allele of Ala64Thr displayed ~2-fold higher risk for CMP compared to GG genotype and G allele, respectively (p<0.05). In Greece, A allele of Ala64Thr decreased risk of CMP by 39%. Healthy individuals with A-3826G GG genotype and carriers of mutant allele of A-112C and Ala64Thr had higher body mass index compared to those carrying other alleles. In healthy Polish, higher waist-to-hip ratio (WHR) was observed in heterozygotes A-3826G compared to AA homozygotes. Heterozygosity of A-112C and Ala64Thr SNPs was related to lower WHR in CMP individuals compared to wild type homozygotes (p<0.05). Meta-analysis showed no statistically significant odds-ratios across our SNPs (p>0.05). Concluding, the studied SNPs could be associated with the most common CMP and their risk factors in some populations.

## Introduction

Single nucleotide polymorphisms (SNPs) in a number of candidate genes are highly implicated in energy balance as well as fat and glucose metabolism, modifying disease susceptibility [1–3]. One of these candidate genes codes for uncoupling protein 1 (UCP1), located on chromosome 4 (4q31.1), which is expressed predominantly in brown adipose tissue, holding a critical role in oxidative phosphorylation and overall energy balance [4, 5]. More than 2300 SNPs have been recognized within the *UCP1* gene and its regulatory regions [6], but four have been commonly studied for their impact on metabolism and energy balance [7–11]. These are: (i) A-3826G (rs1800592) located on the upstream region of *UCP1*, (ii) A-1766G (rs3811791) a 2kb upstream variant, (iii) A-112C (rs10011540) on the 5’UTR region, and (iv) Ala64Thr (rs45539933) a missense variant.

The four *UCP1* SNPs have been associated with a number of cardio-metabolic pathologies (CMP) [12]. The G allele of A-3826G, which is associated with reduced mRNA expression of *UCP1* [13], is more common in obese individuals [14, 15] and it is associated with increased body mass index (BMI), percent body fat, blood pressure [16], and lower high-density lipoprotein level [17]. The same allele of this SNP is associated with higher BMI and glucose levels in overweight persons [18] and can increase the risk for proliferative diabetic retinopathy in individuals with type 2 diabetes [19]. The other three SNPs are less prevalent but have been also associated with various risk factors for CMP [6, 11]. The A-112C polymorphism affects *UCP1* gene promoter activity [20] and the C allele is more frequent in individuals with type 2 diabetes than in healthy individuals [21]. The Ala64Thr mutant allele is associated with higher waist-to-hip ratio (WHR) [22], while the A-1766G SNP, which is detected in the genomic region that possibly regulates transcription of *UCP1* [23], is related with obesity [7]. Finally, the GAA haplotype (A-3826G, A-1766G, and Ala64Thr) is associated with decreased abdominal fat tissue, body fat mass, and WHR [24].

The contribution of the four *UCP1* SNPs to the susceptibility for CMP as well as their involvement in specific risk factors for these conditions varies across populations, even within the same race, probably due to environmental impacts. For instance, the frequency of AG genotype of A-3826G in persons with CMP ranges from 24% in Italy [25], to around 50% in Colombia [20], Japan [21], and Korea [17], and to 85% in China [19]. Similarly, wide frequency ranges have been reported also for the other three SNPs across different populations [10, 20, 26, 27]. At the same time, some studies report that *UCP1* SNPs are strongly associated with disease risk [7, 19, 28], while others report no such findings [29–31]. Therefore, it remains unclear if differences in the prevalence of these four *UCP1* SNPs across different populations are associated with the prevalence of CMP.

Our incomplete understanding about the potential involvement of these four *UCP1* SNPs, among others, in disease susceptibility limits the potential for precision medicine to effectively address CMP. An even more direct effect on disease mitigation is that CMP risk factors are currently addressed with equal importance across different populations, ignoring the genotypic/phenotypic complexity of CMP in different countries. Improving our knowledge about the impact of UCP1 variants can contribute to precision medicine, within the context of approaches that consider the polygenicity of cardio-metabolic traits (e.g., polygenic risk scores). This could improve the sustainability of healthcare systems due to increased efficacy of CMP prevention and mitigation guidelines. To address these important knowledge gaps, we investigated if differences in the frequency of A-3826G, A-1766G, Ala64Thr and A-112C SNPs are associated with the most common CMP and their risk factors. This case control study was performed across five countries (Armenia, Greece, Poland, Russia, United Kingdom) since CMP appear to be increased in certain ethnic groups in Eastern Europe and Western Asia [32, 33].To confirm any observed associations between the studied *UCP1* SNPs and cardio-metabolic health, we extended our findings to consider all previously-studied populations by conducting a systematic review and meta-analysis [34]. The literature includes four meta-analyses [29, 35–37] regarding *UCP1* SNPs and their association with cardio-metabolic traits. Within these four meta-analyses only A-3826G is examined for its association with metabolic diseases or their risk factors, as the most common variant of *UCP1*, while these meta-analyses do not consider the associations of other *UCP1* SNPs with the risk for disease.

## Materials and methods

### Case-control study

This is a multicenter, multinational study conducted during 2016–2019, across five countries (Armenia, Greece, Poland, Russia, and United Kingdom). The participants were recruited via online and paper advertisements as well as word of mouth. Following approval from the relevant Bioethics Review Board in each country (see Section 1.1.1 in S1 File). Written informed consent for participation was signed by the volunteers following detailed explanation of all the procedures and risks involved.

### Study design and data collection

The study involved two groups of participants: individuals with CMP as well as healthy controls. We considered the following CMP, as they present with the highest prevalence [38, 39] amongst all health abnormalities related to cardio-metabolic health: cardiovascular disease, hypertension, metabolic syndrome, and type 2 diabetes. The inclusion criteria were: 1) adult; 2) diagnosed presence of CMP for the CMP group and generally healthy (free of CMP based on their medical history) for the control group; 3) non-smokers, or have quit smoking for at least one year; 4) not in a pregnancy or lactation period; 5) no history of eating disorders; 6) no acute illness and/or infection during the last four weeks.

Ethnicity was self-reported by each participant. All participants were assessed for: 1) medical history via a structured interview-based questionnaire; 2) anthropometry (body height, body mass, WHR); 3) percent fat mass via non-invasive bioelectrical impedance analysis; 4) genotypes of the aforementioned four *UCP1* SNPs detected in DNA isolated from blood samples. A detailed description of the adopted blood handling and genotyping methodologies is provided in Section 1.1.2 in S1 File. All participants were instructed, for 12 hours prior to assessments, to avoid the consumption of food, coffee, or alcohol and to refrain from exercise. Also, they were advised to consume two glasses of water about two hours prior to their assessment.

### Statistical analysis

The data were analyzed using a general genetic model as previously described [40, 41]. We calculated Hardy-Weinberg equilibrium to ensure unbiased outcomes [42]. Linkage disequilibrium between genetic loci, haplotype analysis, and allele frequencies estimation were performed via the SHEsis platform [43, 44]. We used chi-square tests to determine differences in *UCP1* SNPs between groups, as well as Phi indices to report effect sizes [45]. Also, we calculated odds ratios (OR) to determine associations of genotypes and alleles between groups in the overall sample as well as based on country (Section 1.1.3 in S1 File). Finally, we used Kruskal Wallis ANOVA with post hoc Mann-Whitney U tests to assess differences in BMI, WHR, and fat percentage between genotype groups for each *UCP1* SNP. The level of statistical significance for the Hardy-Weinberg equilibrium was set at p<0.05 and for all other analyses at p≤0.05. We did not adjust for multiple comparisons in our study due to the errors and misplaced emphasis associated with such procedures when applied in actual natural observations [46–49].Unless stated otherwise, the SPSS 26.0 (SPSS Inc., Chicago, IL, USA) software was used to perform the statistical analyses.

### Systematic review and meta-analysis

We conducted a systematic review and meta-analysis (PROSPERO review protocol: CRD42019132376) investigating if differences in the frequency of A-3826G, A-1766G, Ala64Thr and A-112C SNPs are associated with the prevalence of the studied CMP. Following the Preferred Reporting Items for Systematic Reviews and Meta-analyses (PRISMA) guidelines [50], we searched the titles and abstracts in PubMed central, Embase, and Cochrane Library (trials) databases from the date of their inception to February 23, 2021, for studies that evaluated the prevalence of *UCP1* A-3826G, A-1766G, Ala64Thr and A-112C SNPs and their association with CMP. No date, participants’ health status, language, or study design limits were applied. A detailed description of the systematic review methodology and the searching algorithm is provided in Section 2.1 in S1 File.

## Results

### Case-control study

#### Associations between genotype frequencies and health status

**Fig 1:**
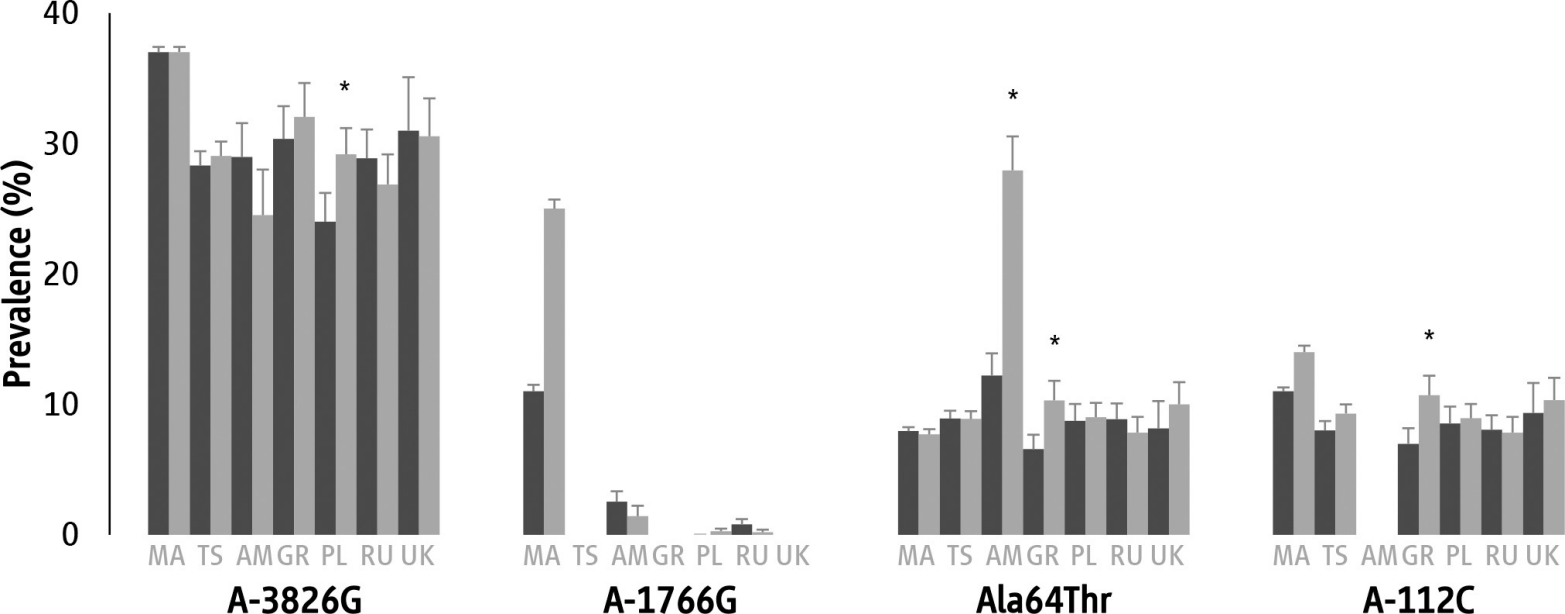
Prevalence of the studied *UCP1* SNP alleles. Note: black bars indicate results for individuals with CMP; gray bars indicate results for healthy persons; * indicates differences from CMP persons significant at p<0.05. Key: MA = meta-analysis, TS = total sample, AM = Armenia, GR = Greece, PL = Poland, RU = Russia, UK = United Kingdom.

The study population included 2283 Caucasian individuals (Table 1). Our Hardy-Weinberg equilibrium (HWE) analysis for the A-1766G revealed significant deviation in healthy individuals (χ2 = 33.34, p<0.001), indicating that this SNP should be excluded from further analysis [42], for other *UCP1* SNPs no deviation from HWE in healthy individuals was noticed. The frequencies of alleles and genotypes for the studied *UCP1* SNPs in healthy controls and in CMP individuals are shown in Fig 1, Table 2 and S4–S11 Tables in S1 File. Odds ratios for the association between genotype and health status (i.e., healthy vs. CMP individuals) for each of the four studied *UCP1* SNPs are shown in Table 2 and S10 and S11 Tables in S1 File.

**Table 1:** Characteristics of the studied population.

	Group	(n) / (%)	Males / Females (n)	Age (years)	BMI (kg/m^2^)
**Entire sample**	Healthy	1139 / 50	762 / 528	45 (32,54)	25.5 (23.9,26.9)
	CMP	1144 / 50	397 / 521	59 (50,65)	30.5 (27.4,34.2)
**Armenia**	Healthy	105 / 32	-	-	-
	CMP	226 / 68	98 / 128	59 (54,64)	29.0 (27.2,31.7)
**Greece**	Healthy	233 / 47	131 / 102	55 (50,65)	26.8 (24.2,29.9)
	CMP	264 / 53	125 / 139	62 (56,68)	31.7 (28.9,34.5)
**Poland**	Healthy	365 / 59	221 /144	32 (25,44)	23.8 (22.0,25.6)
	CMP	252 / 41	89 /163	62 (54.7,67)	31.2 (29.4,33.8)
**Russia**	Healthy	255 / 45	142 / 113	46 (36.5,54.5)	25.9 (25.3,26.3)
	CMP	310 / 55	129 / 181	52 (40,63)	28.9 (26.0,34.6)
**UK**	Healthy	181 / 66	140 / 41	43 (30,51)	25.7 (23.2,29.8)
	CMP	92 / 34	54 / 38	54 (48,57)	30.7 (25.9,38.4)

Note: Age and BMI are presented as median (Q1, Q3).

Key: CMP = cardio-metabolic pathologies; BMI = body mass index; n = number of individuals tested. Q = quartile).

**Table 2:** Frequency of genotypes for Ala64Thr in CMP and healthy individuals.

		Healthy		CMP		OR (95% CI)	F-test
		(n)	(%)	(n)	(%)		
**Total sample**	GG	944	83.39	928	82.71		4.03 p = 0.203
	GA	175	15.46	188	16.76	1.09 (0.87–1.37)	
	AA	13	1.15	6	0.53	0.49 (0.19–1.25)	
	HWE	0.134		0.284			
**Armenia**	GG	90	86.54	164	75.58		5.70 p = 0.031
	GA	14	13.46	53	24.42	2.03 (1.08–3.83)	
	AA	0	0.00	0	0.00	---	
	HWE	0.462		0.040			
**Greece**	GG	184	80.70	219	87.25		4.25 p = 0.115
	GA	41	17.98	31	12.35	0.64 (0.39–1.05)	
	AA	3	1.32	1	0.40	0.36 (0.05–2.46)	
	HWE	0.679		0.931			
**Poland**	GG	304	83.29	211	83.73		0.39 p = 0.842
	GA	58	15.89	38	15.08	0.95 (0.61–1.48)	
	AA	3	0.82	3	1.19	1.44 (0.32–6.40)	
	HWE	0.899		0.394			
**Russia**	GG	218	85.49	257	82.90		1.52 p = 0.454
	GA	34	13.33	51	16.45	1.27 (0.79–2.02)	
	AA	3	1.18	2	0.65	0.61 (0.12–3.10)	
	HWE	0.215		0.758			
**UK**	GG	148	82.22	77	83.70		1.65 p = 0.480
	GA	28	15.56	15	16.30	1.04 (0.53–2.05)	
	AA	4	2.22	0	0.00	0.21 (0.01–4.01)	
	HWE	0.069		0.395			

Key: CMP = cardio-metabolic pathologies; OR = odds ratio; HWE = p value for the Hardy-Weinberg equilibrium.

With regard to country-level stratification, allele frequency analysis (S4–S9 Tables in S1 File) in the Greek population showed that individuals carrying the C allele of the A-112C SNP or the A allele of the Ala64Thr SNP are 37% and 39% less likely to develop CMP, respectively (p<0.05; S6 Table in S1 File). Moreover, the G allele of the A-3826G SNP was associated with 23% lower risk to develop CMP in the Polish population (S7 Table in S1 File).

In total, we found no associations between genotype and health status in the overall sample for the studied *UCP1* SNPs (p>0.05). Though, we observed an association between genotype and health status for Ala64Thr within the Armenian population, where the GA genotype was carried by 24.4% of the CMP individuals but only by 13.5% of healthy individuals. Also, the GA genotype of Ala64Thr showed a 2-fold higher risk (p = 0.03) for CMP than the GG genotype in the Armenian population (Table 2).

#### Linkage disequilibrium

Our analysis for all four SNPs in this study in CMP individuals and healthy controls showed that the A-3826G and Ala64Thr were in strong linkage disequilibrium with a D’ value of 0.831. Similar results were observed for the combinations of A-3826G and A-112C, as well as for the Ala64Thr and A-112C which were in strong linkage disequilibrium with D’ values of 0.917 and 0.924, respectively. However, the r^2^ values for the combinations of A-3826G and Ala64Thr (r^2^ = 0.165) as well as A-3826G and A-112C (r^2^ = 0.195) were relatively low, indicating that their effects are independent of each other. In contrast, the r^2^ value for Ala64Thr and A-112C was high (r^2^ = 0.848), indicating a direct link between these two SNPs. Country-specific analysis of linkage disequilibrium between investigated SNPs can be found in S1 and S2 Figs in S1 File.

#### Haplotype analysis

In the overall sample, the haplotype analysis revealed that CMP individuals were 24% less likely to carry the GAC (A-3826G, Ala64Thr, A-112C) haplotype compared to healthy controls (OR: 0.76 CI95%: 0.60–0.96 p = 0.023; S1 Table in S1 File). Country-specific analysis showed lower CMP risk for this haplotype across countries but this association reached statistical significance only in the Greek population (OR = 0.56, CI95%: 0.34–0.91, p = 0.017). Additionally, in the Polish population, we found a higher frequency of the AGA haplotype in CMP individuals compared to healthy persons (74.9% vs 70.6%), which indicates the relationship between this haplotype and higher risk of CMP (OR = 1.33, CI95%: 1.03–1.73, p = 0.032). On the contrary, for GGA haplotype we found a lower frequency in CMP Polish population compared to healthy individuals (15.6% vs 20.3%) indicating a protective effect in healthy individuals (OR = 0.74, CI95%: 0.55–0.99, p = 0.047). In the Armenian population, the AA haplotype (A-3826G, Ala64Thr) increased the CMP risk more than 4-fold (OR = 4.10, CI95%: 1.12–14.98, p = 0.02), while the AG haplotype decreased the susceptibility to CMP (OR = 0.65, CI95% = 0.45–0.95, p = 0.025). The AA haplotype differs from the AG in the second position defined by the mutant allele of Ala64Thr confirming the association of A allele of this SNP with CMP risk. Detailed results for haplotype analysis for each country are provided in S1 and S2 Tables in S1 File.

#### Association between UCP1 SNPs with specific CMP risk factors

In healthy individuals, we observed significantly higher BMI in the homozygotes GG of A-3826G as compared to AA and AG individuals (p = 0.03) as well as in carriers of the mutant allele of A-112C (p = 0.015), and Ala64Thr (p = 0.004) compared to the wild type homozygotes (Table 3). We also showed that CMP individuals being heterozygotes of A-112C and Ala64Thr had lower WHR than wild type homozygotes (Table 3). Country-specific analysis showed that in the healthy Greek population, heterozygous individuals of A-112C and Ala64Thr displayed higher BMI and fat mass compared to the wild type homozygotes (BMI p = 0.005, body fat p = 0.008 and BMI p = 0.002, body fat p = 0.005, respectively; S14 Table in S1 File). In the Polish healthy population, mutant homozygotes of the A-112C SNP presented higher BMI compared to heterozygotes and wild type homozygotes (S12 Table in S1 File; p<0.05). Due to linkage disequilibrium between A-112C and Ala64Thr, the same effect was observed for mutant homozygotes of Ala64Thr. Finally, in Polish healthy individuals, higher WHR was observed in GA heterozygotes (p = 0.03) in comparison to wild type homozygous subjects (S12 Table in S1 File).

**Table 3:** Body mass index and waist-to-hip ratio [median (Q1, Q3)] across the different *UCP1* SNPs for the entire sample as well as across healthy controls and individuals with CMP.

		Body mass index		Waist-to-hip ratio	
SNP	Genotype	Healthy	CMP	Healthy	CMP
**A-3826G**	AA	25.6 (23.5,26.6)	30.3 (27.4,34.1)^1^	0.87 (0.81,0.93)	0.97 (0.92,1.04)^1^
	AG	25.4 (23.6,27.0)	30.7 (27.5,34.2)^1^	0.88 (0.81,0.93)	1.00 (0.92,1.04)^1^
	GG	26.2 (24.1,28.7)^2^^,^^3^	30.8 (27.2,33.8)^1^	0.88 (0.80,0.92)	1.00 (0.92,1.05)^1^
**A-112C**	AA	25.4 (23.5,26.7)	30.6 (27.5,34.2)^1^	0.87 (0.81,0.93)	0.98 (0.93,1.04)^1^
	AC	25.9 (23.7,28.3)^2^	31.2 (27.3,34.2)^1^	0.88 (0.82,0.94)	0.96 (0.87,1.02)^1^^,^^2^
	CC	26.3 (25.5,27.2)	27.9 (27.3,32.5)^1^	0.87 (0.85,0.89)	0.94 (0.84,1.00)
**Ala64Thr**	GG	25.4 (23.4,26.7)^2^^,^^3^	30.5 (27.4,34.10^1^	0.87 (0.81,0.93)	0.98 (0.93,1.04)^1^^,^^3^
	GA	26.0 (23.8,28.3)	30.5 (27.3,33.7)^1^	0.88 (0.82,0.93)	0.97 (0.87,1.03)^1^
	AA	26.3 (26.1,27.4)	29.8 (27.2,32.7)	0.90 (0.84,0.98)	0.92 (0.80,1.02)

Note

^1^ = difference from healthy significant at p≤0.05

^2^ = difference from AA significant at p≤0.05

^3^ = difference from AG significant at p≤0.05. Key: CMP = cardio-metabolic pathologies

### Systematic review and meta-analysis

#### Searching procedure

The searching procedure retrieved 817 publications of which 109 were duplicates. We excluded 219 publications being reviews, editorials, and conference proceeding as well as 161 publications which referred to animal studies. From the 328 remaining publications, 276 were excluded as they did not meet the inclusion criteria. In total, 52 eligible publications were included in the analysis. Detailed searching procedure results can be found in a PRISMA flowchart (S3 Fig in S1 File).

#### Characteristics of included studies and risk of bias assessment

The 52 eligible publications included in the analysis were published between 1998 and 2020 and included data from 24 different countries. The extracted data for all 52 included publications can be found in S17 Table in S1 File. The risk of bias assessment demonstrated low risk for the vast majority of the eligible studies (Section 2.2 in S1 File).

#### Meta-analysis outcomes

Fifty-one out of the 52 eligible publications [7, 8, 10, 12, 13, 16–21, 25–31, 51–83] were used for prevalence meta-analyses, while 22 eligible publications were used for odds ratios meta-analyses. The results from the meta-analyses are summarized in Fig 1 and Table 4, while the SNP-specific forest and funnel plots for the prevalence (S5–S24 and S35–S44 Figs in S1 File) and the odds ratios (S25–S34 and S45–S49 Figs in S1 File) can be found in Sections 2.2.1 and 2.2.2 in S1 File. On the whole, for the different genotypes and alleles we performed 24 prevalence meta-analyses and 12 odds ratios meta-analyses which included a total of 34,313 cases. No statistically significant differences were observed in the prevalence of the mutant alleles of the four different SNPs (p>0.05; Fig 1). Also, when we considered only case-control studies, we found no statistically significant odds ratios in different alleles across the four studied SNPs (p>0.05).

**Table 4:** Meta-analysis results for the prevalence and odds ratios of genotypes of the four different SNPs, between healthy and CMP individuals.

SNP	n	Genotypes	Prevalence meta-analyses		OR meta-analyses	
			Healthy (%)	CMP (%)	OR (95%CI)	p
A-3826G	18568	AA	43	42		
		AG	43	43	1.02 (0.96–1.09)	0.46
		GG	14	15	1.06 (0.96–1.17)	0.23
A-112C	6153	AA	77	78		
		AC	21	21	1.07 (0.80–1.44)	0.65
		CC	2	1	0.92 (0.65–1.32)	0.67
Ala64Thr	4984	GG	85	82		
		GA	14	17	1.07 (0.91–1.27)	0.41
		AA	1	1	0.64 (0.24–1.67)	0.36
A-1766G	4608	AA	64	66		
		AG	30	29	1.12 (0.81–1.55)	0.51
		GG	6	5	1.04 (0.53–2.04)	0.90

Key: CMP = cardio-metabolic pathologies; n = number of studied individuals; OR = odds ratio with reference to AA; 95%CI = 95% confidence intervals; p = p value for the Z test indicating the overall effect in the meta-analysis.

## Discussion

Our findings confirm an association between the studied *UCP1* SNPs and cardiometabolic health in a multi-country sample of 2,283 persons. Furthermore, we found that differences in the distribution of genotypes and alleles of the studied SNPs between CMP individuals and healthy controls are associated with the prevalence of one or more of the most common CMP and their risk factors, in some (Armenia, Greece, and Poland) but not all (Russia and United Kingdom) countries.

Within our study population, the A-3826G (AG) was the most prevalent of the four SNPs. In persons with CMP, the prevalence was 40%, ranging from 34% in the UK to 42% in Armenia and Russia. This is very similar to the 43% found in our meta-analysis, and mid-way between the 29% reported in Spain [16] and the ~50% reported in Colombia [8], Japan [21], and Korea [17]. Our findings in the case-control study indicate that the A-3826G is not associated with CMP, but that it leads to increased BMI within the healthy population. Thus, it may promote the development of CMP in the presence of environmental factors [84] as well as other genetic traits [85].

Our results for Ala64Thr and A-112C indicate a strong linkage disequilibrium between the two SNPs. In our study the mutant A allele of Ala64Thr was detected in 9% of both healthy individuals and persons with CMP, and this frequency was not very different across the five studied countries. This was similar to the 7% for healthy and 9% for CMP individuals found in our meta-analysis that included data from 4984 persons across nine countries. Our observed prevalence rates for the C allele of A-112C were 9% in healthy persons and 8% in individuals with CMP. This was somewhat lower than the 12% prevalence found in our meta-analysis that included data from 6,153 persons across eight countries. In terms of health impacts, we showed that the Ala64Thr and A-112C are associated with opposing effects in healthy individuals and persons with CMP. Our results indicate that the A-112C mutant allele demonstrates its effect when present in its heterozygous form and this may be the reason for C allele’s association with decreased risk for CMP development. Specifically, we found that healthy individuals carrying the mutant alleles display higher BMI and, in some countries, body fat percent. On the other hand, persons with CMP who carry the mutant variants have lower WHR. These results partly reflect those reported in previous studies [22, 24]. For instance, the presence of mutant alleles Ala64Thr and A-1766G, in combination with A-3826G, can augment the beneficial effects of caloric restriction resulting in greater reductions in WHR [22]. Unfortunately, we were not able to assess potential associations of these SNPs with biochemical indices or with additional clinical features.

It is important to consider the functional impact of A-3826G, A-1766G and Ala64Thr, which is clear since they directly affect the expression of *UCP1*. In the case of A-112C, it is important to also consider the effect of another variant, rs72941746, that is in linkage disequilibrium [86]. The A-112C seems to modify 4 transcription factor binding sites and its region has specific patterns of chromatin accessibility in several tissues. It appears that the linked variant is responsible for much more alterations in transcription factor binding site motifs and consequently the binding of other proteins. This indicates that the association observed in this study when A-112C is present could possibly be an effect of rs72941746 influence.

Our findings indicate potential limitations of common analysis of different races, ethnicities, and regions when analyzing our data as an entire sample or via meta-analytic methods. For instance, the frequency of A allele of Ala64Thr across all our studied countries was 9%, similar to the 8% found in our meta-analysis, in both cases suggesting no differences between healthy persons and individuals with CMP. However, our country-specific analysis demonstrated that the prevalence of A allele of Ala64Thr was significantly higher in healthy individuals across the Armenian (27.9%) and the Greek (10.3%) populations, as compared to CMP persons. Considering risk factors, we detected a number of associations with the four studied SNPs across Greece, Armenia and Poland, which were not observed in the other countries. Taken together, these findings suggest that the studied SNPs may be important for promoting risk factors and pathophysiological mechanisms involved in CMP, but that this involvement may be stronger in some races, ethnicities, and/or regions. Nevertheless, it is important to also note that the increased CMP prevalence in certain ethnic groups in Eastern Europe and Western Asia [32, 33] may reflect potential ancestral differential effects. While we made every effort to achieve representativeness and increase our sample sizes, we acknowledge that labeling of ancestral populations by self-reported ethnicity does not fully account for genetic variations.

Our results may reflect that ethnicity was self-determined by the participants and potential relationships between them were not investigated. This approach may not always reflect the inter/intra ethnic variation in the frequency distribution of germline variants of the population examined. Also, we were unable to explore additional factors associated with CMPs, including demographic characteristics (socioeconomic status, etc.) and environmental factors (climate conditions, nutritional habits, etc.).

We conclude that, in some populations, the A-3826G, A-1766G, Ala64Thr and A-112C SNPs of *UCP1* gene may be associated with the prevalence of one or more of the most common CMP and their risk factors. Future studies on these SNPs may shed more light on the genetics of CMP and may uncover potential candidates for precision medicine.

## Supporting information

S1 Checklist Meta-analysis on genetic association studies checklist. (DOCX)

S1 File Detailed results and analyses from the case-control study as well as the systematic review and meta-analysis. (DOCX)

## References

[1] Groop L. Genetics of the metabolic syndrome. Br J Nutr. 2000;83 Suppl 1:S39–48. doi: 10.1017/s0007114500000945 . 10889791

[2] Mirkov S, Myers JL, Ramírez J, Liu W. SNPs affecting serum metabolomic traits may regulate gene transcription and lipid accumulation in the liver. Metabolism: clinical and experimental. 2012;61(11):1523–7. doi: 10.1016/j.metabol.2012.05.004 22738862 PMC3867007

[3] Shastry BS. SNP alleles in human disease and evolution. J Hum Genet. 2002;47(11):561–6. Epub 2002/11/19. doi: 10.1007/s100380200086 . 12436191

[4] Dinas PC, Valente A, Granzotto M, Rossato M, Vettor R, Zacharopoulou A, et al. Browning formation markers of subcutaneous adipose tissue in relation to resting energy expenditure, physical activity and diet in humans. Horm Mol Biol Clin Investig. 2017;31(1). doi: 10.1515/hmbci-2017-0008 . 28678735

[5] Valente A, Jamurtas AZ, Koutedakis Y, Flouris AD. Molecular pathways linking non-shivering thermogenesis and obesity: focusing on brown adipose tissue development. Biol Rev Camb Philos Soc. 2015;90(1):77–88. doi: 10.1111/brv.12099 . 24708171

[6] Flouris AD, Dinas PC, Valente A, Andrade CMB, Kawashita NH, Sakellariou P. Exercise-induced effects on UCP1 expression in classical brown adipose tissue: a systematic review. Horm Mol Biol Clin Investig. 2017;31(2). doi: 10.1515/hmbci-2016-0048 . 28085671

[7] Chathoth S, Ismail MH, Vatte C, Cyrus C, Al Ali Z, Ahmed KA, et al. Association of Uncoupling Protein 1 (UCP1) gene polymorphism with obesity: a case-control study. BMC Med Genet. 2018;19(1):203. doi: 10.1186/s12881-018-0715-5 ; PubMed Central PMCID: PMC6247512. 30458724 PMC6247512

[8] Franco-Hincapie L, Duque CE, Parra MV, Gallego N, Villegas A, Ruiz-Linares A, et al. Association between polymorphism in uncoupling proteins and type 2 diabetes in a northwestern Colombian population. Biomedica. 2009;29(1):108–18. . 19753844

[9] Jia JJ, Tian YB, Cao ZH, Tao LL, Zhang X, Gao SZ, et al. The polymorphisms of UCP1 genes associated with fat metabolism, obesity and diabetes. Mol Biol Rep. 2010;37(3):1513–22. Epub 2009/05/16. doi: 10.1007/s11033-009-9550-2 . 19444646

[10] Lim JH, Ko MM, Moon TW, Cha MH, Lee MS. Association of the UCP-1 single nucleotide polymorphism A-3826G with the dampness-phlegm pattern among Korean stroke patients. BMC Complement Altern Med. 2012;12:180. Epub 2012/10/10. doi: 10.1186/1472-6882-12-180 ; PubMed Central PMCID: PMC3537753. 23043591 PMC3537753

[11] Pravednikova AE, Shevchenko SY, Kerchev VV, Skhirtladze MR, Larina SN, Kachaev ZM, et al. Association of uncoupling protein (Ucp) gene polymorphisms with cardiometabolic diseases. Molecular medicine (Cambridge, Mass). 2020;26(1):51. Epub 2020/05/27. doi: 10.1186/s10020-020-00180-4 ; PubMed Central PMCID: PMC7249395 or financial relationships that could be construed as a potential conflict of interest. 32450815 PMC7249395

[12] Brondani LA, Assmann TS, Duarte GC, Gross JL, Canani LH, Crispim D. The role of the uncoupling protein 1 (UCP1) on the development of obesity and type 2 diabetes mellitus. Arq Bras Endocrinol Metabol. 2012;56(4):215–25. Epub 2012/07/14. doi: 10.1590/s0004-27302012000400001 . 22790465

[13] Esterbauer H, Oberkofler H, Liu YM, Breban D, Hell E, Krempler F, et al. Uncoupling protein-1 mRNA expression in obese human subjects: the role of sequence variations at the uncoupling protein-1 gene locus. J Lipid Res. 1998;39(4):834–44. Epub 1998/04/29. . 9555947

[14] Hayakawa T, Nagai Y, Taniguchi M, Yamashita H, Takamura T, Abe T, et al. Phenotypic characterization of the beta3-adrenergic receptor mutation and the uncoupling protein 1 polymorphism in Japanese men. Metabolism. 1999;48(5):636–40. doi: 10.1016/s0026-0495(99)90063-x . 10337866

[15] Ramis JM, Gonzalez-Sanchez JL, Proenza AM, Martinez-Larrad MT, Fernandez-Perez C, Palou A, et al. The Arg64 allele of the beta 3-adrenoceptor gene but not the -3826G allele of the uncoupling protein 1 gene is associated with increased leptin levels in the Spanish population. Metabolism. 2004;53(11):1411–6. doi: 10.1016/j.metabol.2004.06.006 . 15536594

[16] Forga L, Corbalan M, Marti A, Fuentes C, Martinez-Gonzalez MA, Martinez A. Influence of the polymorphism 03826 A—> G in the UCP1 gene on the components of metabolic syndrome. An Sist Sanit Navar. 2003;26(2):231–6. doi: 10.23938/ASSN.0449 . 12951617

[17] Oh HH, Kim KS, Choi SM, Yang HS, Yoon Y. The effects of uncoupling protein-1 genotype on lipoprotein cholesterol level in Korean obese subjects. Metabolism. 2004;53(8):1054–9. Epub 2004/07/29. doi: 10.1016/j.metabol.2004.02.014 . 15281018

[18] Heilbronn LK, Kind KL, Pancewicz E, Morris AM, Noakes M, Clifton PM. Association of -3826 G variant in uncoupling protein-1 with increased BMI in overweight Australian women. Diabetologia. 2000;43(2):242–4. Epub 2001/02/07. doi: 10.1007/s001250050036 . 10753048

[19] Zhang Y, Meng N, Lv Z, Li H, Qu Y. The gene polymorphisms of UCP1 but not PPAR γ and TCF7L2 are associated with diabetic retinopathy in Chinese type 2 diabetes mellitus cases. Acta Ophthalmol. 2015;93(3):e223–9. Epub 2014/10/03. doi: 10.1111/aos.12542 . 25274455

[20] Fukuyama K, Ohara T, Hirota Y, Maeda K, Kuno S, Zenibayashi M, et al. Association of the -112A>C polymorphism of the uncoupling protein 1 gene with insulin resistance in Japanese individuals with type 2 diabetes. Biochem Biophys Res Commun. 2006;339(4):1212–6. Epub 2005/12/13. doi: 10.1016/j.bbrc.2005.11.140 . 16338218

[21] Mori H, Okazawa H, Iwamoto K, Maeda E, Hashiramoto M, Kasuga M. A polymorphism in the 5’ untranslated region and a Met229—>Leu variant in exon 5 of the human UCP1 gene are associated with susceptibility to type II diabetes mellitus. Diabetologia. 2001;44(3):373–6. Epub 2001/04/25. doi: 10.1007/s001250051629 . 11317671

[22] Herrmann SM, Wang JG, Staessen JA, Kertmen E, Schmidt-Petersen K, Zidek W, et al. Uncoupling protein 1 and 3 polymorphisms are associated with waist-to-hip ratio. J Mol Med (Berl). 2003;81(5):327–32. Epub 2003/05/21. doi: 10.1007/s00109-003-0431-1 . 12756473

[23] Soo Kim K, Cho D-Y, Joo Kim Y, Choi SM, Kim JY, Shin SU, et al. The finding of new genetic polymorphism of UCP-1 A-1766G and its effects on body fat accumulation. Biochimica et Biophysica Acta (BBA)—Molecular Basis of Disease. 2005;1741(1):149–55. 10.1016/j.bbadis.2004.11.026 15955458

[24] Shin HD, Kim KS, Cha MH, Yoon Y. The effects of UCP-1 polymorphisms on obesity phenotypes among Korean female subjects. Biochem Biophys Res Commun. 2005;335(2):624–30. doi: 10.1016/j.bbrc.2005.07.096 . 16084837

[25] Montesanto A, Bonfigli AR, Crocco P, Garagnani P, De Luca M, Boemi M, et al. Genes associated with Type 2 Diabetes and vascular complications. Aging (Albany NY). 2018;10(2):178–96. doi: 10.18632/aging.101375 ; PubMed Central PMCID: PMC5842840. 29410390 PMC5842840

[26] Pei X, Liu L, Cai J, Wei W, Shen Y, Wang Y, et al. Haplotype-based interaction of the PPARGC1A and UCP1 genes is associated with impaired fasting glucose or type 2 diabetes mellitus. Medicine (Baltimore). 2017;96(23):e6941. Epub 2017/06/08. doi: 10.1097/MD.0000000000006941 ; PubMed Central PMCID: PMC5466206. 28591028 PMC5466206

[27] Vimaleswaran KS, Radha V, Ghosh S, Majumder PP, Rao MR, Mohan V. A haplotype at the UCP1 gene locus contributes to genetic risk for type 2 diabetes in Asian Indians (CURES-72). Metab Syndr Relat Disord. 2010;8(1):63–8. Epub 2009/12/01. doi: 10.1089/met.2009.0039 . 19943796

[28] Cha MH, Kang BK, Suh D, Kim KS, Yang Y, Yoon Y. Association of UCP1 genetic polymorphisms with blood pressure among Korean female subjects. Journal of Korean medical science. 2008;23(5):776–80. Epub 2008/10/29. doi: 10.3346/jkms.2008.23.5.776 ; PubMed Central PMCID: PMC2580006. 18955781 PMC2580006

[29] de Souza BM, Brondani LA, Bouças AP, Sortica DA, Kramer CK, Canani LH, et al. Associations between UCP1 -3826A/G, UCP2 -866G/A, Ala55Val and Ins/Del, and UCP3 -55C/T polymorphisms and susceptibility to type 2 diabetes mellitus: case-control study and meta-analysis. PLoS One. 2013;8(1):e54259. Epub 2013/02/01. doi: 10.1371/journal.pone.0054259 ; PubMed Central PMCID: PMC3554780. 23365654 PMC3554780

[30] Malczewska-Malec M, Wybranska I, Leszczynska-Golabek I, Partyka L, Hartwich J, Jabrocka A, et al. Analysis of candidate genes in Polish families with obesity. Clin Chem Lab Med. 2004;42(5):487–93. Epub 2004/06/19. doi: 10.1515/CCLM.2004.083 . 15202783

[31] Schaffler A, Palitzsch KD, Watzlawek E, Drobnik W, Schwer H, Scholmerich J, et al. Frequency and significance of the A—>G (-3826) polymorphism in the promoter of the gene for uncoupling protein-1 with regard to metabolic parameters and adipocyte transcription factor binding in a large population-based Caucasian cohort. Eur J Clin Invest. 1999;29(9):770–9. doi: 10.1046/j.1365-2362.1999.00529.x . 10469165

[32] Balkau B, Deanfield JE, Després J-P, Bassand J-P, Fox KAA, Smith SC Jr., et al. International Day for the Evaluation of Abdominal Obesity (IDEA): a study of waist circumference, cardiovascular disease, and diabetes mellitus in 168,000 primary care patients in 63 countries. Circulation. 2007;116(17):1942–51. doi: 10.1161/CIRCULATIONAHA.106.676379 . 17965405 PMC2475527

[33] WHO. Global Atlas on Cardiovascular Disease Prevention and Control. Geneva: World Health Organization;World Heart Federation; World Stroke Organization. 2011.

[34] Higgins JP, Thomas J. Cochrane Handbook for Systematic Reviews of Interventions: Cochrane collaboration 2019.

[35] Brondani LA, Assmann TS, de Souza BM, Boucas AP, Canani LH, Crispim D. Meta-analysis reveals the association of common variants in the uncoupling protein (UCP) 1–3 genes with body mass index variability. PLoS One. 2014;9(5):e96411. doi: 10.1371/journal.pone.0096411 24804925 PMC4013025

[36] de Almeida Brondani L, de Souza BM, Assmann TS, Bouças AP, Bauer AC, Canani LH, et al. Association of the UCP polymorphisms with susceptibility to obesity: case–control study and meta-analysis. Mol Biol Rep. 2014;41(8):5053–67. doi: 10.1007/s11033-014-3371-7 24752406

[37] Liu X, Jiang Z, Zhang G, Ng TK, Wu Z. Association of UCP1 and UCP2 variants with diabetic retinopathy susceptibility in type-2 diabetes mellitus patients: A meta-analysis. BMC Ophthalmol. 2021;21(1):1–12. doi: 10.1186/s12886-020-01714-4 33579234 PMC7881628

[38] Cardiovascular diseases (CVDs) [Internet]. 2017 [cited 6 May 2020]. Available from: https://www.who.int/en/news-room/fact-sheets/detail/cardiovascular-diseases-(cvds)

[39] Noncommunicable diseases [Internet]. 2018 [cited 6 May 2020]. Available from: https://www.who.int/news-room/fact-sheets/detail/noncommunicable-diseases

[40] Clarke GM, Anderson CA, Pettersson FH, Cardon LR, Morris AP, Zondervan KT. Basic statistical analysis in genetic case-control studies. Nat Protoc. 2011;6(2):121–33. doi: 10.1038/nprot.2010.182 21293453 PMC3154648

[41] Lunetta KL. Genetic association studies. Circulation. 2008;118(1):96–101. Epub 2008/07/02. doi: 10.1161/CIRCULATIONAHA.107.700401 . 18591452

[42] Namipashaki A, Razaghi-Moghadam Z, Ansari-Pour N. The Essentiality of Reporting Hardy-Weinberg Equilibrium Calculations in Population-Based Genetic Association Studies. Cell J. 2015;17(2):187–92. Epub 2015/07/23. doi: 10.22074/cellj.2016.3711 ; PubMed Central PMCID: PMC4503832. 26199897 PMC4503832

[43] Li Z, Zhang Z, He Z, Tang W, Li T, Zeng Z, et al. A partition-ligation-combination-subdivision EM algorithm for haplotype inference with multiallelic markers: update of the SHEsis (http://analysis.bio-x.cn). Cell Res. 2009;19(4):519–23. Epub 2009/03/18. doi: 10.1038/cr.2009.33 . 19290020

[44] Shi YY, He L. SHEsis, a powerful software platform for analyses of linkage disequilibrium, haplotype construction, and genetic association at polymorphism loci. Cell Res. 2005;15(2):97–8. Epub 2005/03/03. doi: 10.1038/sj.cr.7290272 . 15740637

[45] Kim H-Y. Statistical notes for clinical researchers: Chi-squared test and Fisher’s exact test. Restorative dentistry & endodontics. 2017;42(2):152–5. Epub 2017/03/30. doi: 10.5395/rde.2017.42.2.152 . 28503482 PMC5426219

[46] Feise RJ. Do multiple outcome measures require p-value adjustment? BMC Med Res Methodol. 2002;2:8. Epub 2002/06/19. doi: 10.1186/1471-2288-2-8 ; PubMed Central PMCID: PMC117123. 12069695 PMC117123

[47] Perneger TV. What’s wrong with Bonferroni adjustments. BMJ. 1998;316(7139):1236–8. Epub 1998/05/16. doi: 10.1136/bmj.316.7139.1236 ; PubMed Central PMCID: PMC1112991. 9553006 PMC1112991

[48] Rothman KJ. No adjustments are needed for multiple comparisons. Epidemiology. 1990;1(1):43–6. Epub 1990/01/01. . 2081237

[49] Rothman KJ. Six persistent research misconceptions. J Gen Intern Med. 2014;29(7):1060–4. Epub 2014/01/24. doi: 10.1007/s11606-013-2755-z ; PubMed Central PMCID: PMC4061362. 24452418 PMC4061362

[50] Moher D, Liberati A, Tetzlaff J, Altman DG, Group P. Preferred reporting items for systematic reviews and meta-analyses: the PRISMA statement. PLoS Med. 2009;6(7):e1000097. Epub 2009/07/22. doi: 10.1371/journal.pmed.1000097 ; PubMed Central PMCID: PMC2707599. 19621072 PMC2707599

[51] Bracale R, Labruna G, Finelli C, Daniele A, Sacchetti L, Oriani G, et al. The absence of polymorphisms in ADRB3, UCP1, PPARγ, and ADIPOQ genes protects morbid obese patients toward insulin resistance. Journal of endocrinological investigation. 2012;35(1):2–4. Epub 2012/03/07. doi: 10.1007/BF03345413 . 22391136

[52] Brondani LA, de Souza BM, Assmann TS, Bouças AP, Bauer AC, Canani LH, et al. Association of the UCP polymorphisms with susceptibility to obesity: case-control study and meta-analysis. Mol Biol Rep. 2014;41(8):5053–67. Epub 2014/04/23. doi: 10.1007/s11033-014-3371-7 . 24752406

[53] Brondani LA, Duarte GC, Canani LH, Crispim D. The presence of at least three alleles of the ADRB3 Trp64Arg (C/T) and UCP1 -3826A/G polymorphisms is associated with protection to overweight/obesity and with higher high-density lipoprotein cholesterol levels in Caucasian-Brazilian patients with type 2 diabetes. Metab Syndr Relat Disord. 2014;12(1):16–24. Epub 2013/10/22. doi: 10.1089/met.2013.0077 . 24138564

[54] Chen Y, Wang X, Shen Z, Fan P, Liu R, Liu Y, et al. Effect of the beta-3 adrenergic receptor Trp64Arg and uncoupling protein 1–3826 A>G genotypes on lipid and apolipoprotein levels in overweight/obese and non-obese Chinese subjects. Lipids Health Dis. 2015;14:34. Epub 2015/05/01. doi: 10.1186/s12944-015-0029-y ; PubMed Central PMCID: PMC4410578. 25928572 PMC4410578

[55] Csernus K, Pauler G, Erhardt É, Lányi É, Molnár D. Effects of energy expenditure gene polymorphisms on obesity-related traits in obese children. Obes Res Clin Pract. 2015;9(2):133–40. Epub 2014/08/02. doi: 10.1016/j.orcp.2014.06.001 . 25081806

[56] Dhall M, Chaturvedi MM, Rai U, Kapoor S. Sex-dependent effects of the UCP1–3826 A/G polymorphism on obesity and blood pressure. Ethn Dis. 2012;22(2):181–4. Epub 2012/07/07. . 22764640

[57] Dong C, Lv Y, Xie L, Yang R, Chen L, Zhang L, et al. Association of UCP1 polymorphisms with type 2 diabetes mellitus and their interaction with physical activity and sedentary behavior. Gene. 2020;739:144497. Epub 2020/02/24. doi: 10.1016/j.gene.2020.144497 . 32088243

[58] Gagnon J, Lago F, Chagnon YC, Pérusse L, Näslund I, Lissner L, et al. DNA polymorphism in the uncoupling protein 1 (UCP1) gene has no effect on obesity related phenotypes in the Swedish Obese Subjects cohorts. Int J Obes Relat Metab Disord. 1998;22(6):500–5. Epub 1998/07/17. doi: 10.1038/sj.ijo.0800613 . 9665669

[59] Hamada T, Kotani K, Nagai N, Tsuzaki K, Matsuoka Y, Sano Y, et al. Low-calorie diet-induced reduction in serum HDL cholesterol is ameliorated in obese women with the -3826 G allele in the uncoupling protein-1 gene. Tohoku J Exp Med. 2009;219(4):337–42. Epub 2009/12/08. doi: 10.1620/tjem.219.337 . 19966534

[60] Jin P, Li Z, Xu X, He J, Chen J, Xu X, et al. Analysis of association between common variants of uncoupling proteins genes and diabetic retinopathy in a Chinese population. BMC Med Genet. 2020;21(1):25. Epub 2020/02/08. doi: 10.1186/s12881-020-0956-y ; PubMed Central PMCID: PMC7006419. 32028915 PMC7006419

[61] Kieć-Wilk B, Wybrańska I, Malczewska-Malec M, Leszczyńska-Gołabek L, Partyka L, Niedbał S, et al. Correlation of the -3826A >G polymorphism in the promoter of the uncoupling protein 1 gene with obesity and metabolic disorders in obese families from southern Poland. J Physiol Pharmacol. 2002;53(3):477–90. Epub 2002/10/12. . 12375583

[62] Kotani K, Fujiwara S, Tsuzaki K, Sano Y, Nagai N, Yamada T, et al. The Association Between the Uncoupling Protein-1 Gene A-3826G Polymorphism and High-density Lipoprotein Cholesterol in A General Japanese Population: A Consideration of the Obesity Status. J Clin Med Res. 2011;3(6):319–24. Epub 2012/03/07. doi: 10.4021/jocmr738w ; PubMed Central PMCID: PMC3279477. 22393344 PMC3279477

[63] Kotani K, Sakane N, Saiga K, Adachi S, Shimohiro H, Mu H, et al. Relationship between A-3826G polymorphism in the promoter of the uncoupling protein-1 gene and high-density lipoprotein cholesterol in Japanese individuals: a cross-sectional study. Arch Med Res. 2008;39(1):142–6. Epub 2007/12/11. doi: 10.1016/j.arcmed.2007.07.002 . 18068010

[64] Labruna G, Pasanisi F, Nardelli C, Tarantino G, Vitale DF, Bracale R, et al. UCP1–3826 AG+GG genotypes, adiponectin, and leptin/adiponectin ratio in severe obesity. J Endocrinol Invest. 2009;32(6):525–9. Epub 2009/05/29. doi: 10.1007/BF03346500 . 19474520

[65] Lin E, Pei D, Huang YJ, Hsieh CH, Wu LS. Gene-gene interactions among genetic variants from obesity candidate genes for nonobese and obese populations in type 2 diabetes. Genet Test Mol Biomarkers. 2009;13(4):485–93. Epub 2009/07/15. doi: 10.1089/gtmb.2008.0145 . 19594364

[66] Lindholm E, Klannemark M, Agardh E, Groop L, Agardh CD. Putative role of polymorphisms in UCP1-3 genes for diabetic nephropathy. J Diabetes Complications. 2004;18(2):103–7. Epub 2004/05/04. doi: 10.1016/S1056-8727(03)00019-9 . 15120704

[67] Mottagui-Tabar S, Hoffstedt J, Brookes AJ, Jiao H, Arner P, Dahlman I. Association of ADRB1 and UCP3 gene polymorphisms with insulin sensitivity but not obesity. Horm Res. 2008;69(1):31–6. Epub 2007/12/07. doi: 10.1159/000111793 . 18059082

[68] Nakatochi M, Ushida Y, Yasuda Y, Yoshida Y, Kawai S, Kato R, et al. Identification of an interaction between VWF rs7965413 and platelet count as a novel risk marker for metabolic syndrome: an extensive search of candidate polymorphisms in a case-control study. PLoS One. 2015;10(2):e0117591. Epub 2015/02/04. doi: 10.1371/journal.pone.0117591 ; PubMed Central PMCID: PMC4315519 following conflicts: the authors TN and MI are employees of Toyota Motor Co., Ltd. This does not alter the authors’ adherence to all the PLOS ONE policies on sharing data and materials. 25646961 PMC4315519

[69] Nicoletti CF, de Oliveira AP, Brochado MJ, de Oliveira BP, Pinhel MA, Marchini JS, et al. UCP1–3826 A>G polymorphism affects weight, fat mass, and risk of type 2 diabetes mellitus in grade III obese patients. Nutrition. 2016;32(1):83–7. Epub 2015/10/16. doi: 10.1016/j.nut.2015.07.016 . 26458326

[70] Nieters A, Becker N, Linseisen J. Polymorphisms in candidate obesity genes and their interaction with dietary intake of n-6 polyunsaturated fatty acids affect obesity risk in a sub-sample of the EPIC-Heidelberg cohort. Eur J Nutr. 2002;41(5):210–21. Epub 2002/10/24. doi: 10.1007/s00394-002-0378-y . 12395215

[71] Proenza AM, Poissonnet CM, Ozata M, Ozen S, Guran S, Palou A, et al. Association of sets of alleles of genes encoding beta3-adrenoreceptor, uncoupling protein 1 and lipoprotein lipase with increased risk of metabolic complications in obesity. Int J Obes Relat Metab Disord. 2000;24(1):93–100. Epub 2000/03/07. doi: 10.1038/sj.ijo.0801091 . 10702757

[72] Rudofsky G Jr., Schrödter A, Voron’ko OE, Schlotterer A, Humpert PM, Tafel J, et al. Promoter polymorphisms of UCP1, UCP2, and UCP3 are not associated with diabetic microvascular complications in type 2 diabetes. Horm Metab Res. 2007;39(4):306–9. Epub 2007/04/21. doi: 10.1055/s-2007-973816 . 17447170

[73] Rudofsky G Jr., Schroedter A, Schlotterer A, Voron’ko OE, Schlimme M, Tafel J, et al. Functional polymorphisms of UCP2 and UCP3 are associated with a reduced prevalence of diabetic neuropathy in patients with type 1 diabetes. Diabetes Care. 2006;29(1):89–94. Epub 2005/12/24. doi: 10.2337/diacare.29.01.06.dc05-0757 . 16373902

[74] Sale MM, Hsu FC, Palmer ND, Gordon CJ, Keene KL, Borgerink HM, et al. The uncoupling protein 1 gene, UCP1, is expressed in mammalian islet cells and associated with acute insulin response to glucose in African American families from the IRAS Family Study. BMC Endocr Disord. 2007;7:1. Epub 2007/04/03. doi: 10.1186/1472-6823-7-1 ; PubMed Central PMCID: PMC1852562. 17397545 PMC1852562

[75] Sámano R, Huesca-Gómez C, López-Marure R, Hernández-Cabrera AK, Rodríguez-Ventura A, Tolentino M, et al. Association between UCP polymorphisms and adipokines with obesity in Mexican adolescents. J Pediatr Endocrinol Metab. 2018;31(5):561–8. Epub 2018/04/11. doi: 10.1515/jpem-2017-0262 . 29634487

[76] Sivenius K, Valve R, Lindi V, Niskanen L, Laakso M, Uusitupa M. Synergistic effect of polymorphisms in uncoupling protein 1 and beta3-adrenergic receptor genes on long-term body weight change in Finnish type 2 diabetic and non-diabetic control subjects. Int J Obes Relat Metab Disord. 2000;24(4):514–9. Epub 2000/05/11. doi: 10.1038/sj.ijo.0801194 . 10805511

[77] Sramkova D, Krejbichova S, Vcelak J, Vankova M, Samalikova P, Hill M, et al. The UCP1 gene polymorphism A-3826G in relation to DM2 and body composition in Czech population. Exp Clin Endocrinol Diabetes. 2007;115(5):303–7. Epub 2007/05/23. doi: 10.1055/s-2007-977732 . 17516293

[78] Sun H, Zhang JT, Xie XR, Li T, Li XY, Wang NN, et al. Association of uncoupling protein gene polymorphisms with essential hypertension in a northeastern Han Chinese population. J Hum Hypertens. 2019;33(7):524–30. Epub 2018/12/07. doi: 10.1038/s41371-018-0141-3 . 30518806

[79] Tiwari AK, Prasad P, B KT, Kumar KM, Ammini AC, Gupta A, et al. Oxidative stress pathway genes and chronic renal insufficiency in Asian Indians with Type 2 diabetes. J Diabetes Complications. 2009;23(2):102–11. Epub 2008/04/17. doi: 10.1016/j.jdiacomp.2007.10.003 . 18413200

[80] Verdi H, Kınık ST, Baysan-Çebi HP, Yalçın YY, Yazıcı-Güvercin AC, Aydın B, et al. Uncoupling protein gene UCP1-3826A/G, UCP2 Ins/Del and UCP3-55C/T polymorphisms in obese Turkish children. Turk J Pediatr. 2020;62(6):921–9. Epub 2020/12/30. doi: 10.24953/turkjped.2020.06.003 . 33372430

[81] Vimaleswaran KS, Radha V, Deepa R, Mohan V. Absence of Association of Metabolic Syndrome with PPARGC1A, PPARG and UCP1 Gene Polymorphisms in Asian Indians. Metab Syndr Relat Disord. 2007;5(2):153–62. Epub 2008/03/29. doi: 10.1089/met.2006.0032 . 18370824

[82] Yiew SK, Khor LY, Tan ML, Pang CL, Chai VY, Kanachamy SS, et al. No association between peroxisome proliferator-activated receptor and uncoupling protein gene polymorphisms and obesity in Malaysian university students. Obes Res Clin Pract. 2010;4(4):e247–342. Epub 2010/10/01. doi: 10.1016/j.orcp.2010.03.002 . 24345699

[83] Zietz B, Watzlawek E, Palitzsch KD, Schölmerich J, Schäffler A. GG-genotype in the promotor region of uncoupling-protein-1 gene is associated with lower level of dehydroepiandrosterone in type 2 diabetes. Exp Clin Endocrinol Diabetes. 2001;109(2):102–6. Epub 2001/05/09. doi: 10.1055/s-2001-14829 . 11341297

[84] Schwartz DA. Environmental genomics and human health. G Ital Med Lav Ergon. 2011;33(1):31–4. Epub 2011/03/23. . 21417136

[85] Yoneshiro T, Ogawa T, Okamoto N, Matsushita M, Aita S, Kameya T, et al. Impact of UCP1 and β3AR gene polymorphisms on age-related changes in brown adipose tissue and adiposity in humans. Int J Obes. 2013;37(7):993–8. doi: 10.1038/ijo.2012.161 23032405

[86] Ward LD, Kellis M. HaploReg v4: systematic mining of putative causal variants, cell types, regulators and target genes for human complex traits and disease. Nucleic Acids Res. 2016;44(D1):D877–D81. Epub 2015/12/10. doi: 10.1093/nar/gkv1340 . 26657631 PMC4702929

