# Supplementary material for: Prevalence of uncoupling protein one genetic polymorphisms and their relationship with cardiovascular and metabolic health
Source: PLoS One. 2022 Apr 28;17(4):e0266386. doi: 10.1371/journal.pone.0266386 (PMC9049362; doi:10.1371/journal.pone.0266386)
Supplement: S1 Checklist — (DOCX) [file pone.0266386.s001.docx]

**Meta-analysis on Genetic Association Studies Checklist | PLOS ONE**

|  | Item | Section name and paragraph number within manuscript |
| --- | --- | --- |
|  | **Introduction** |  |
| 1 | Provide a detailed justification for the polymorphism studied; if a single polymorphism was analyzed, give details as to why others were not included in the meta-analysis. | 1.Introduction, paragraphs 1,2,3 |
| 2 | Provide a detailed justification for the population(s) and clinical condition studied. | 1. Introduction, paragraph 4 |
|  | **Methods** |  |
| 3 | Provide full details of the search strategy employed; outline the full electronic search strategy –specific combination of keywords and any limits applied- for at least one database. Specify whether synonyms of polymorphisms/genes (e.g. SNP number) were searched. | 2.2 Systematic Review and meta-analysis, paragraph 1 and S 2.1.1. Search strategy, selection criteria and meta-analysis process |
| 4 | Report full details on the inclusion and exclusion criteria applied for selecting studies.  *Please list the excluded articles and the reasons for exclusion of each article in a supplementary file.* | 2.2 Systematic Review and metanalysis, paragraph 1 and S 2.2.1, paragraph 2 |
| 5 | Provide details on how the quality of the studies included in the analyses was assessed. | 3.2.2 Characteristic of included studies and risk of bias assessment and S 2.1.1. Search strategy, selection criteria and meta-analysis process , paragraph 2 |
| 6 | Describe steps taken to contact study authors to identify additional studies and to request missing data. | We have identified an adequate number of eligible studies (n=52) to test our research question and therefore, no exhaustive searching approach was used- |
| 7 | Describe how environmental effects were adjusted for, if this adjustment was not conducted, outline the reasons for this. | Environmental effects were not adjusted, since these varieties were tested via the heterogeneity index - |
| 8 | Describe the methods of handling heterogeneity/between-study variance. | S 2.1.1. Search strategy, selection criteria and meta-analysis process |
| 9 | Describe how the Hardy-Weinberg equilibrium and linkage disequilibrium were assessed. | , paragraph 3 |
| 10 | Describe and justify the choice of model for the analyses (per-allele vs per-genotype vs genetic model-free, random effects vs fixed effects). | S 2.1.1. Search strategy, selection criteria and meta-analysis process, paragraph 3 |
| 11 | Describe whether a sensitivity analysis has been completed. | Sensitivity analysis was not required to test our research question- |
| 12 | Describe whether an assessment of the effects of population stratification has been conducted. | The population was stratified according to our research question (cardiometabolic pathologies and healthy) - |
| 13 | Describe whether study-specific results have been assessed and if so the reasons for this (e.g. forest plot). | 2.2.1, paragraph 1 |
|  | **Results** |  |
| 14 | Include flow diagram for the studies included in the meta-analysis as the first figure for the manuscript | The flow diagram is provided in the supplement, Figure S3- |
| 15 | Report details on allele/genotype prevalence. | 3.2.3 Metanalysis outcomes, paragraph 1 |
| 16 | Report the effect size estimates and p values for each analysis. | 3.2.3 Metanalysis outcomes, paragraph 1 |
|  | **Discussion** |  |
| 17 | Discuss the limitations of the meta-analysis, including genotyping errors/bias and publication bias. | Limitations are included in the discussion (line 311)- |
| 18 | If the meta-analysis identifies an association within a subgroup of the population studied but not another, discuss the implications of these results, and if applicable the possibility of subgroup-specific publication bias. | 4. Discussion, paragraph 3,4 |
| 19 | Discuss the suitability of the sample size employed to the research question and the power of the study. | 4. Discussion, paragraph 3,4 |
