# Supplementary material for: Prevalence of uncoupling protein one genetic polymorphisms and their relationship with cardiovascular and metabolic health
Source: PLoS One. 2022 Apr 28;17(4):e0266386. doi: 10.1371/journal.pone.0266386 (PMC9049362; doi:10.1371/journal.pone.0266386)
Supplement: Attachment — Submitted filename: Response to Reviewers.docx [file pone.0266386.s003.docx]

Dear Reviewers,

We wish to thank you for reviewing our work. We understand that the limitations identified during the review process were important and we appreciate the fact that you valued the interest of our paper and have given us a chance to revise it.

We made appropriate changes in the manuscript based on the comments that we received, and we believe that the revisions have markedly improved the paper’s quality. Our point-by-point responses to the Reviewers’ comments appear below. Red font is used to indicate revised parts of the text.

Since the present is a revised version of a previously-submitted manuscript, we submitted a copy of the paper where the changes/modifications based on the comments appear as track changes.

**JOURNAL REQUIREMENTS:**

**Comment 1:** Please ensure that your manuscript meets PLOS ONE's style requirements, including those for file naming. The PLOS ONE style templates can be found at

https://journals.plos.org/plosone/s/file?id=wjVg/PLOSOne_formatting_sample_main_body.pdf and

https://journals.plos.org/plosone/s/file?id=ba62/PLOSOne_formatting_sample_title_authors_affiliations.pdf

**Response to Comment 2:** Done as requested.

**Comment 2:** Please include additional information regarding the survey or questionnaire used in the study and ensure that you have provided sufficient details that others could replicate the analyses. For instance, if you developed a questionnaire as part of this study and it is not under a copyright more restrictive than CC-BY, please include a copy, in both the original language and English, as Supporting Information.

**Response to Comment 2:** We did not use a survey or questionnaire in the study.

**Comment 3:** We note that the grant information you provided in the ‘Funding Information’ and ‘Financial Disclosure’ sections do not match.

When you resubmit, please ensure that you provide the correct grant numbers for the awards you received for your study in the ‘Funding Information’ section.

**Response to Comment 3:** Done as requested.

**Comment 4:** 4. We note that you have provided additional information within the Acknowledgements Section that is not currently declared in your Funding Statement. Please note that funding information should not appear in the Acknowledgments section or other areas of your manuscript. We will only publish funding information present in the Funding Statement section of the online submission form.

Please remove any funding-related text from the manuscript and let us know how you would like to update your Funding Statement. Currently, your Funding Statement reads as follows:

NO

Please include your amended statements within your cover letter; we will change the online submission form on your behalf.

**Response to Comment 4:** The funding support for this study now appears clearly in the cover letter, the manuscript, and the online submission system, and is as follows:

- European Union 7th Framework Program (FP7-PEOPLE-2013-IRSES Grant No. 319010; U-GENE project
- Russian Science Foundation grant 20-14-00201 (case-control study in the Russian population; meta-analysis).
- Polish Ministry of Science and Higher Education 2016-2017 international project co-financed W15/7.PR/2016.

**Comment 5:** Thank you for stating the following in your Competing Interests section:

NO authors have competing interests

Please complete your Competing Interests on the online submission form to state any Competing Interests. If you have no competing interests, please state "The authors have declared that no competing interests exist.", as detailed online in our guide for authors at http://journals.plos.org/plosone/s/submit-now

This information should be included in your cover letter; we will change the online submission form on your behalf.

**Response to Comment 5:** We clearly state in our cover letter that “The authors have declared that no competing interests exist.” Thank you for changing this in the online submission form on our behalf.

**Comment 6:** In your Data Availability statement, you have not specified where the minimal data set underlying the results described in your manuscript can be found. PLOS defines a study's minimal data set as the underlying data used to reach the conclusions drawn in the manuscript and any additional data required to replicate the reported study findings in their entirety. All PLOS journals require that the minimal data set be made fully available. For more information about our data policy, please see http://journals.plos.org/plosone/s/data-availability.

Upon re-submitting your revised manuscript, please upload your study’s minimal underlying data set as either Supporting Information files or to a stable, public repository and include the relevant URLs, DOIs, or accession numbers within your revised cover letter. For a list of acceptable repositories, please see http://journals.plos.org/plosone/s/data-availability#loc-recommended-repositories. Any potentially identifying patient information must be fully anonymized.

Important: If there are ethical or legal restrictions to sharing your data publicly, please explain these restrictions in detail. Please see our guidelines for more information on what we consider unacceptable restrictions to publicly sharing data: http://journals.plos.org/plosone/s/data-availability#loc-unacceptable-data-access-restrictions. Note that it is not acceptable for the authors to be the sole named individuals responsible for ensuring data access.

We will update your Data Availability statement to reflect the information you provide in your cover letter.

**Response to Comment 6:** We have now made the data for our study freely available in Figshare. The following statement has been included in the manuscript, the cover letter, and the online submission form:

“Datasets related to this study have been uploaded on Figshare (https://figshare.com/articles/dataset/UGENE_project_DATABASE_All_participants_xlsx/17206709) under DOI: 10.6084/m9.figshare.17206709.”

**Comment 7:** Please include captions for your Supporting Information files at the end of your manuscript, and update any in-text citations to match accordingly. Please see our Supporting Information guidelines for more information: http://journals.plos.org/plosone/s/supporting-information.

**Response to Comment 7:** Done as requested.

**REVIEWER #1**

**Comment 8:** In this manuscript, Dinas and co-workers carried out a population-based genetic epidemiology study, aimed to document the frequency of four UCP1 gene polymorphisms and to examine its association with cardiovascular-metabolic health in 2283 multiethnic European individuals. Besides, the authors have carried out a meticulous meta-analysis cum systemic review to extend their findings. Overall, the manuscript is well written, and the attempt of the authors should be praised. However, there are some significant issues in the manuscript that need to be addressed.

**Response to Comment 8:** Thank you for your supportive comments. We made every effort to address your comments and our point-by-point responses appear below.

**Comment 9:** The authors are advised to revise the language of the manuscript, as some passages are inaccurate or contain semantic and syntactic mistakes. Further, there are several typos and spacing issues in the manuscript (for example: on line 86, the word ‘relat-ed’, on line 154 and 212, the word ‘be-tween’, on line 236, the word ‘statis-tical’, on line 360 ‘SNPs UCP1 SNPs’etc). This needs to be addressed.

**Response to Comment 9:** Thank you for this comment. Typos and spacing issues have now been resolved throughout the text. A fluent English speaker has also screened the manuscript for grammatical errors.

**Comment 10:** The most important result of the study might have also been influenced by a selection bias. How were the probands chosen? How do the authors define an individual as Russian, Armenian ethnic, and healthy? This is a population-based genetic epidemiological study, and it is important to briefly describe the influence of ethnicity/race on inter/intra ethnic variation in the frequency distribution of germline variants.

**Response to Comment 10:** We agree with the Reviewer that genetic epidemiological studies can be influenced by selection bias. Therefore, we applied the following changes in order to clarify this important aspect as suggested.

Line 117-123 (Introduction): “To address these important knowledge gaps, we investigated if differences in the frequency of A-3826G, A-1766G, Ala64Thr and A-112C SNPs are associated with the most common CMP and their risk factors. This case control study was performed across five countries (Armenia, Greece, Poland, Russia, United Kingdom) since CMPs appear to be increased in certain ethnic groups in Eastern Europe and Western Asia [32, 33].”

Line 153 (Methods): “Ethnicity was self-reported by each participant.”

Line 392-395 (Discussion): “Our results may reflect that ethnicity was self-determined by the participants and potential relationships between them were not investigated. This approach may not always reflect the inter/intra ethnic variation in the frequency distribution of germline variants of the population examined.”

Also, we added the following clarification with respect to how we defined health in the control group:

Line 147-152 (Methods): “The inclusion criteria were: 1) adult; 2) diagnosed presence of CMP for the CMP group and generally healthy (free of CMP based on their medical history) for the control group; 3) non-smokers, or have quit smoking for at least one year; 4) not in a pregnancy or lactation period; 5) no history of eating disorders; 6) no acute illness and/or infection during the last four weeks.”

**Comment 11:**  I was wondering why there is no data on the clinical and biochemical features of the study subjects. And whether multivariable analysis to evaluate independent associations has been performed. Further, the paper is assessing several associations facing the risks of multiple testing. Correction is warranted.

**Respond to Comment 11:** Thank you for this comment. As indicated in the text (Lines 154-157), some clinical features were evaluated (i.e. BMI, WHR, fat mass percentage) and the results from these analyses are described in the Results and Discussion. Unfortunately, we were not able to assess biochemical features. These issues have been now included as a limitation of our study:

Line 360-361 (Discussion): “Unfortunately, we were not able to assess potential associations of these SNPs with biochemical indices or with additional clinical features.”

Regarding the suggested adjustment to the alpha level, we chose to not correct for multiple comparisons in our study. There have been a number of papers which conclude that such a procedure leads to significant errors of interpretation. Rothman (Epidemiology 1990; 1:43-46) noted that “a policy of not making adjustments for multiple comparisons is preferable because it will lead to fewer errors of interpretation when the data under evaluation are not random numbers but actual observations on nature”. The major misconceptions associated with this procedure were recently highlighted in a paper by the same author (Rothman, J Gen Intern Med. 2014 Jan 23.) as well as others (Perneger 1998; Feise 2002). As noted in the 2014 paper by Rothman “if one is analyzing biological data that are replete with actual associations, the premise for traditional adjustments are shaky and the adjustments are difficult to defend”. In addition to the above, as indicated by Reviewer 2, a correction of false positives may be too strict for our study which uses a small number of SNPs and they show linkage between them.

For the above reasons, we did not include an adjustment in our analyses. Nevertheless, we do appreciate that readers should be aware of this. Therefore, we made relevant insertions in the Statistical Analysis and the Discussion sections, as follows:

Line 176-178 (Statistical Analysis): “We did not adjust for multiple comparisons in our study due to the errors and misplaced emphasis associated with such procedures when applied in actual natural observations [46-49].”

**Comment 12:** Add a note in the introduction/methods stating the putative function of the studied SNPs (alters expression, alters binding site, etc), chromosome location, and position. Further, the author should comment on the possible molecular mechanism by which the studied polymorphisms influence the disease risk.

**Respond to Comment 12:** Thank you for this constructive comment. We agree that this information is essential in the text. Therefore, we introduced the following changes in the text:

Line 66-69 (Introduction): One of these candidate genes codes for uncoupling protein 1 (UCP1), located on chromosome 4 (4q31.1), which is expressed predominantly in brown adipose tissue, holding a critical role in oxidative phosphorylation and overall energy balance [4, 5].

Line 76-92 (Introduction): “The four *UCP1* SNPs have been associated with a number of cardio-metabolic pathologies (CMP) [12]. The G allele of A-3826G, which is associated with reduced mRNA expression of *UCP1* [13], is more common in obese individuals [14, 15] and it is associated with increased body mass index (BMI), percent body fat, blood pressure [16], and lower high-density lipoprotein level [17]. The same allele of this SNP is associated with higher BMI and glucose levels in overweight persons [18] and can increase the risk for proliferative diabetic retinopathy in individuals with type 2 diabetes [19]. The other three SNPs are less prevalent but have been also associated with various risk factors for CMP [6, 11]. The A-112C polymorphism affects *UCP1* gene promoter activity [20] and the C allele is more frequent in individuals with type 2 diabetes than in healthy individuals [21]. The Ala64Thr mutant allele is associated with higher waist-to-hip ratio (WHR) [22], while the A-1766G SNP, which is detected in the genomic region that possibly regulates transcription of *UCP1* [23], is related with obesity [7]. Finally, the GAA haplotype (A-3826G, A-1766G, and Ala64Thr) is associated with decreased abdominal fat tissue, body fat mass, and WHR [24].

**Comment 13:** What is the time span for the study recruitment? Did they validate the genotyping methodology? If yes, please mention it. Also, the authors need to elaborate on whether a phenotype-blind genotyping process was pursued.

**Response to Comment 13:** Thank you for noting the absence of this important information, considering the validation of the genotyping method, the timespan of the recruitment and the blinding process. We have now filled in the missing information by adding the following text:

Line 134 (Methods): “This is a multicenter, multinational study conducted during 2016-2019 across five countries…”

Supplement Section 1.1.2 “For all the analyzed samples, a phenotype-blind genotyping process was adopted. Validation of the genotyping methodology in all the countries has been performed either by direct sequencing of random samples or by PCR-restriction fragment length polymorphism (PCR-RFLP) method as described below.”

Supplement Section 1.1.2a: “To perform the quality control of the genotyping method, we assessed PCR-products of randomly chosen samples from each genotype by direct sequencing.”

**Comment 14:** Several other demographic/environmental factors and social determinants were not explored in this study which could potentially affect the associations. Do the authors think that this study has any limitations? It is missing in the discussion section.

**Response to Comment 14:** Indeed, this was an important omission. We added several limitations of the study to appropriately inform readers. These include the lack of investigating other factors and social determinants that could potentially affect the associations. The relevant paragraph in the Discussion section is provided below:

Line 392-398 (Discussion): “Our results may reflect that ethnicity was self-determined by the participants and potential relationships between them were not investigated. This approach may not always reflect the inter/intra ethnic variation in the frequency distribution of germline variants of the population examined. Also, we were unable to explore additional factors associated with CMP, including demographic characteristics (socioeconomic status, etc.) and environmental factors (climate conditions, nutritional habits, etc.).”

We also added the following limitations in other parts of the Discussion:

Line 360-361 (Discussion): “Unfortunately, we were not able to assess potential associations of these SNPs with biochemical indices or with additional clinical features.”

Line 385-391 (Discussion): “Nevertheless, it is important to also note that the increased CMP prevalence in certain ethnic groups in Eastern Europe and Western Asia [32, 33] may reflect potential ancestral differential effects. While we made every effort to achieve representativeness and increase our sample sizes, we acknowledge that labeling of ancestral populations by self-reported ethnicity does not fully account for genetic variations.”

**Comment 15:** What about the association analysis of UCP1 gene polymorphisms with CMP risk, stratified by gender?

**Response to Comment 15:** We agree with the Reviewer that this is an important research question to investigate. We plan to present these results in a separate paper. Including these results in the current paper would dilute the main message and would add twice more content and tables in an already data-heavy article with an 80-page Supplement. We hope that the Reviewer will agree with this approach.

**Comment 16:** Potential ancestral differential effects should be discussed more as they present a considerable contribution to the study. Throughout the manuscript, the gene names should be in italics.

**Response to Comment 16:** We added extensive comments on the potential effects of ancestral differences, as follows:

Line 385-391 (Discussion): “Nevertheless, it is important to also note that the increased CMP prevalence in certain ethnic groups in Eastern Europe and Western Asia [32, 33] may reflect potential ancestral differential effects. While we made every effort to achieve representativeness and increase our sample sizes, we acknowledge that labeling of ancestral populations by self-reported ethnicity does not fully account for genetic variations.”

Gene names have now been placed in italics throughout the text.

**REVIEWER #2**

**Comment 17:** The authors performed a genetic association study to test the influence of genetic variants of UCP1 on cardiometabolic pathologies (CMP) and their risk factors. They included Caucasian individuals from different populations. UCP1 is a key protein in the function of brown adipose tissue, which is implicated in the non-shivering thermogenesis. The authors found different allele frequencies between cases (CMP) and controls for some UCP1 SNPs in some populations. In addition, some alleles/genotypes were associated with CMP risk factors. I think this study can be relevant for improving our understanding of the genetic architecture of CMP and the role of UCP1. However, I have some comments and concerns.

**Response to Comment 17:** Thank you for your supportive comments. We made every effort to address your comments and our point-by-point responses appear below.

**Comment 18:** Line 67: Missing comma before "which".

**Response to Comment 18:** The comma has now been added.

**Comment 19:** Lines 76-78: The G allele of A-3826G associates with higher HDL? This would mean less cardiometabolic risk.

**Response to Comment 19:** Thank you for noticing this mistake. The text has now been corrected as follows:

Line 79-80: “…and it is associated with increased body mass index (BMI), percent body fat, blood pressure [16], and lower high-density lipoprotein level [17].”

**Comment 20:** Line 86: "related" instead of "relat-ed".

**Response to Comment 20:** Typo corrected.

**Comment 21:** Line 92: do you mean the frequency of the GENOTYPE AG among persons with CMP? Maybe add "AG genotype".

**Response to Comment 21:** The AG genotype has now been added.

Line 96: “For instance, the frequency of AG genotype of A-3826G in persons…”

**Comment 22:** Lines 102-107: I agree with the point that a better knowledge of the genetic architecture of CMP can help in their prevention and treatment, but this statement focused on UCP proteins is too strong. Cardiometabolic traits are complex, being influenced by many genes with small effect sizes. Improving our knowledge about the impact of UCP1 variants can contribute to precision medicine, but within the context of polygenic scores considering thousands of other variants. It is unlikely that these common variants can have practical relevance in isolation. Please, modify and soften this statement.

**Response to Comment 22:** Thank you for your constructive comment. We agree that our statement considering *UCP1* SNPs was too strong. Thus, after your recommendation we have modified the text to soften this statement, as follows:

Line 105-117 (Introduction): “Our incomplete understanding about the potential involvement of these four *UCP1* SNPs, among others, in disease susceptibility limits the potential for precision medicine to effectively address CMP. An even more direct effect on disease mitigation is that CMP risk factors are currently addressed with equal importance across different populations, ignoring the genotypic/phenotypic complexity of CMPs in different countries. Improving our knowledge about the impact of *UCP1* variants can contribute to precision medicine, within the context of polygenic scores considering thousands of other variants. This could improve the sustainability of healthcare systems due to increased efficacy of CMP prevention and mitigation guidelines.”

**Comment 23:** There is any control for population structure? I understand that you performed analyses within each region, removing the influence of genetic clusters across Europe. However, structure could also exist within a population. You had evidence that all individuals in each group belonged to the same race/ethnicity and individuals were not related? If not, please clearly state this as limitation of the study.

**Response to Comment 23:** We agree with the Reviewer that structures within our populations may have influenced our results. Therefore, we applied the following changes to clarify this important aspect, as suggested:

Line117-123 (Introduction): “To address these important knowledge gaps, we investigated if differences in the frequency of A-3826G, A-1766G, Ala64Thr and A-112C SNPs are associated with the most common CMP and their risk factors. This case control study was performed across five countries (Armenia, Greece, Poland, Russia, United Kingdom) since CMPs appear to be increased in certain ethnic groups in Eastern Europe and Western Asia [32, 33].”

Line 153 (Methods): “Ethnicity was self-reported by each participant.”

Line 392-395 (Discussion): “Our results may reflect that ethnicity was self-determined by the participants and potential relationships between them were not investigated. This approach may not always reflect the inter/intra ethnic variation in the frequency distribution of germline variants of the population examined.”

**Comment 24:** I do not fully understand table 1.1.3 in supplementary. The prevalence is the result of dividing cases with a given genotype by the total sample size. Then, the prevalence should be between 0 and 1. Maybe the prevalence is shown in brackets, while the actual number of genotype carriers is show above? Please, clarify.

**Response to Comment 24:** Thank you for noticing. We have now corrected the legend of S1 and S2 Tables to be more explanative for the reader, as follows:

“S1 Table: Haplotype frequency [n (prevalence)] for UK, Greece…”

“S2 Table: Haplotype frequency [n (prevalence)] in Armenia…”

**Comment 25:** I am not very familiar with the methodology of meta-analyses, this could be also the case for other readers. Could you extend the explanations and rationale of the decisions made? Several choices were made based on previous guidelines. Maybe you could explain briefly the rationale in the supplementary.

**Response to Comment 25:** We added the following information in the supplement to expand on the rationale for the choices made in our meta-analyses:

Section 2.2.1:

“**Meta-analysis Methodology**

The aim of the systematic review and meta-analysis study was to investigate whether differences in the frequency of A-3826G, A-1766G, Ala64Thr and A-112C SNPs are associated with the most common CMP and their risk factors, in the existing already published literature. An important index to calculate these differences in frequencies, is the prevalence index. Therefore, we conducted prevalence meta-analyses by dividing the incidence of CMP by the overall sample size [a (incidence of genotype)/b (sample size)] of each study for UCP1 A-3826G, A-1766G, Ala64Thr and A-112C SNPs. These meta-analyses were conducted for each one of the UCP1 homozygous and heterozygous genotypes as well as for the mutant alleles of each studied SNP. Standard errors for these meta-analyses were calculated using the following formula: a (incidence of genotype/allele) /[a (incidence of genotype/allele) *b (sample size)]2. Standard errors were then used for weighted proportions and the RevMan 5.3 software [4] to generate forest and funnel plots. Another important index to test whether there is an association between a group of CMP individuals and a group of healthy participants in the UCP1 homozygous and heterozygous genotypes as well as mutant alleles, is the odds ratio. Therefore, we conducted odds ratio meta-analyses, using a dichotomous, inverse variance, random-effect model, via the RevMan 5.3 software.4 Incidence of each one of the UCP1 homozygous and heterozygous genotypes and mutant alleles were calculated between a group of CMP individuals and a group of healthy participants, while weighted proportions were calculated based on each study’s sample size. For all meta-analyses, we evaluated the 95% confidence interval (CI) and heterogeneity between studies using the I² statistic. Heterogeneity is an index that tests any kind of variability (i.e. effect estimate) among the included studies in a systematic review and meta-analysis.[5] We considered a statistically significant result for heterogeneity when p<0.10, while interpretation of I2 index was made based on previous guidelines.[5] A heterogeneity of 0%-40% might not be important, 30%-60% may represent moderate heterogeneity, 50%-90% may represent substantial heterogeneity and 75%-100% represents considerable heterogeneity.[5]”

**Comment 26:** Figure 1: I would avoid the colors in Figure 1. It makes difficult the visualization of the patterns. I think it is enough with the country name at the bottom. I would also add a legend in the plot reminding that patterned bars are "healthy" and solid bars are "CMP".

**Response to Comment 26:** As requested, we have now converted the figure to black and white format.

**Comment 27:** I have not seen any control for multiple comparison. When multiple tests are performed, the risk of false positives accumulates across the different P<0.05 tests performed, increasing the probability of type I error. I am aware that this study is using just a few SNPs and they show linkage between them, which decreases the number of independent tests. Under that scenario, a stringent control of false positives like the Bonferroni correction would be too stringent. However, I think the authors could use other approaches less stringent, like the False Discovery Rate. They are considering several populations and phenotypes, so the total number of tests is not low. At minimum, a mention to this aspect should be done in the Discussion, indicating that a control for increased false positive rate has not been done despite the multiple tests performed.

**Response to Comment 27:** We added a brief comment on this issue in the text. Please, see our response to comment 11 by Reviewer 1.

**Comment 28:** In line with the previous comment about the risk of false positives, I think it would be good to add some additional evidence about the functional relevance of the variants. The impact of Ala64Thr is clear, but what about variants from non-coding regions? For example, A-112C seems to modify 4 transcription factor binding sites and its region has specific patterns of chromatin accessibility in several tissues (i.e., DNAse). However, a SNP in linkage disequilibrium (rs72941746) alters much more transcription motifs and more proteins binds in that position (from Haploref, see link below). This could be the causal variant behind the associations of A-112C. Indeed, RegulomeD gives a score of 0.81 to this variant for its support as functional, while A-112C gets 0.61 (the score ranges from 0 to 1). I think additional support coming from functional data can be relevant, specially considering the negative results in the meta-analysis.
- <https://pubs.broadinstitute.org/mammals/haploreg/haploreg.php>
- <https://regulomedb.org/regulome-summary/?regions=rs10011540%0D%0Ars72941746&genome=GRCh37&maf=0.01>

**Response to Comment 28:** Thank you for your comment. We agree that information on the functional relevance of our SNPs is required. Thus, we have now added the following text:

Line 362-371 (Discussion): “It is important to consider the functional impact of A-3826G, A-1766G and Ala64Thr, which is clear since they directly affect the expression of UCP1. In the case of A-112C, it is important to also consider the effect of another variant, rs72941746, that is in linkage disequilibrium [86]. The A-112C seems to modify 4 transcription factor binding sites and its region has specific patterns of chromatin accessibility in several tissues. It appears that the linked variant is responsible for much more alterations in transcription motifs and consequently the binding of other proteins. This indicates that the association observed in this study when A-112C is present could possibly be an effect of rs72941746 influence.”

**Comment 29:** line 260: "compared" instead of "com-pared". There are several typos like this through the manuscript. Please, check it.

**Response to Comment 29:** Thank you for noticing this. Typos have now been corrected throughout the text.

**Comment 30:** Individuals of the Greek population carrying the C allele of A-112C are 37% less likely to develop CMP. However, healthy individuals with the AC genotype have higher BMI than AA carriers for the entire sample. Do you have any potential explanation for this pattern? You mention in Discussion the existence of contrasting patterns between healthy and CMP groups, but I do not see a convincing explanation.

**Response to Comment 30:** Thank you for your comment. We added the following comment in response to the point that you raised:

Line 348-353 (Discussion): “In terms of health impacts, we showed that the Ala64Thr and A-112C are associated with opposing effects in healthy individuals and persons with CMP. Our results indicate that the A-112C mutant allele demonstrates its effect when present in its heterozygous form and this may be the reason for C allele’s association with decreased risk for CMP development.”
