# Supplementary material for: Prevalence of uncoupling protein one genetic polymorphisms and their relationship with cardiovascular and metabolic health
Source: PLoS One. 2022 Apr 28;17(4):e0266386. doi: 10.1371/journal.pone.0266386 (PMC9049362; doi:10.1371/journal.pone.0266386)
Supplement: Attachment — Submitted filename: Response to Reviewers R2.docx [file pone.0266386.s004.docx]

**Reviewer #1:**

**Comment 1:** In this revised manuscript, the authors have satisfactorily responded to all the concerns from the previous review and made tremendous improvements in the revision.

**Response to Comment 1:** Thank you for your supportive comments. We made every effort to address your comments and our point-by-point responses appear below.

**Comment 2:** On line 246-247: the haplotype analysis revealed that CMP individuals were 24% less likely to carry the GAC haplotype, what do you mean by?

**Response to Comment 2:** Thank you for your comment. Indeed, we have not appropriately described the statistical relationship of the reported values. Thus, we have now changed the text as follows:

Line 243: “In the overall sample, the haplotype analysis revealed that CMP individuals were 24% less likely to carry the GAC (A-3826G, Ala64Thr, A-112C) haplotype compared to healthy controls (OR: 0.76 CI95%: 0.60-0.96 p=0.023; S1 Table).”

**Comment 3:** On line 400: It should be written as ‘the A-3826G, A-1766G, Ala64Thr and A-112C SNPs of UCP1 gene’

**Response to Comment 3:** Thank you for noticing. The changes have been applied as requested.

Line 396-397: “We conclude that, in some populations, the A-3826G, A-1766G, Ala64Thr and A-112C SNPs of UCP1 gene may be associated with...”

**Reviewer #2**

**Comment 4:** In general, the authors has addressed the comments well. I have just a few specific comments about the revision.

**Response to Comment 4:** Thank you for your supportive comments. We made every effort to address your comments and our point-by-point responses appear below.

**Comment 5:** Line 114: As a modification of my own previous comment, it would be better to say: "Improving our knowledge about the impact of UCP1 variants can contribute to precision medicine, within the context of approaches that consider the polygenicity of cardio-metabolic traits (e.g., polygenic risk scores)."

**Response to Comment 5:** Thank you, we modified the text according to your recommendation.

Line 109-110: “Improving our knowledge about the impact of UCP1 variants can contribute to precision medicine, within the context of approaches that consider the polygenicity of cardio-metabolic traits (e.g., polygenic risk scores).”

**Comment 6:** Line 176: I do not agree with the argumentation made by the authors. I do think that the risk of false positives and false negatives should be considered depending on the question and the goal. Despite this, I respect their decision of not doing it. This has to be stated in the manuscript, as they have already done, so the reader can take this aspect into account when interpreting the results.

**Response to Comment 6:** Thank you for your constructive comment. We agree that the readers should take this aspect into account when interpreting the results, and for this reason we have added the relevant text.

**Comment 7:** Line 330: Strange wording: "the A-3826G (AG) was the most prevalent of the four SNPs studied in persons with CMP was 40%,". I guess the prevalence was 40% overall. Please, modify.

**Response to Comment 7:** Thank you for noticing. Indeed, the text was difficult to read, thus we modified it as follows:

Line 327: “Within our study population, the A-3826G (AG) was the most prevalent of the four SNPs. In persons with CMP, prevalence was 40%, ranging from 34% in the UK to 42% in Armenia and Russia.”
